# Thin Magnetically Permeable Targets for Inductive Sensing: Application to Limb Prosthetics

**DOI:** 10.3390/s19184041

**Published:** 2019-09-19

**Authors:** Ethan J. Weathersby, Clement J. Gurrey, Jake B. McLean, Benjamin N. Sanders, Brian G. Larsen, Ryan Carter, Joseph L. Garbini, Joan E. Sanders

**Affiliations:** Department of Bioengineering, University of Washington, 3720 15th Ave NE, Box 355061, Seattle, WA 98195-5061, USA; ethanw3@uw.edu (E.J.W.); cgurrey@uw.edu (C.J.G.); bglars@uw.edu (B.G.L.); rvcarter@uw.edu (R.C.); garbini@uw.edu (J.L.G.)

**Keywords:** amputee, inductive sensor, residual limb, transtibial, prosthesis, limb-to-socket distance, adjustable socket

## Abstract

The purpose of this research was to create a thin ferrous polymer composite to be used as a target for inductive sensing in limb prosthetics. Inductive sensors are used to monitor limb-to-socket distance in prosthetic sockets, which reflects socket fit. A styrene–ethylene–ethylene/propylene–styrene (SEEPS) polymer was mixed with iron powder at three concentrations (75, 77, 85 wt%), and thin disk-shaped samples were fabricated (0.50, 0,75, 1.00 mm thickness). For 85 wt% samples of 0.50 mm thickness, which proved the best combination of high signal strength and low target volume, inductive sensor sensitivity ranged from 3.2E5 counts/mm at 0.00–1.00 mm distances to 7.2E4 counts/mm at 4.00–5.00 mm distances. The application of compressive stress (up to 425 kPa) introduced an absolute measurement error of less than 3.3 μm. Tensile elasticity was 282 kPa, which is comparable to that of commercial elastomeric liners. Durability testing in the shoe of an able-bodied participant demonstrated a change in calibration coefficient of less than 3.8% over two weeks of wear. The ferrous polymer composite may facilitate the development of automatically adjusting sockets that use limb-to-socket distance measurement for feedback control.

## 1. Introduction

Residual limb volume loss is a common problem faced by people using prosthetic limbs [1]. Over the course of the day, when people with limb amputation lose limb volume their socket loosens. Changes in the amount of fluid within the residual limb (e.g., interstitial fluid) are the primary source of diurnal limb volume fluctuation. Loose prosthetic sockets may create excessive motion at the limb-socket interface and put prosthesis users at risk of falling. They may also cause focused interface stresses that induce tissue injury. The time course of injury varies among individuals, ranging from minutes to weeks. People with comorbidities such as diabetes and peripheral arterial disease are considered at greater risk and are prone to breakdown sooner than people who had their amputation as a result of trauma without secondary complications.

Prosthesis users are advised to add prosthetic socks to compensate for volume loss, a process that many people with limb amputation find inconvenient and time-consuming. Prosthesis users wearing pants must remove them to effect sock addition. Some people choose to avoid the inconvenience and tolerate a poor socket fit, which may restrict their activity and lead to a worsened disability.

Sockets designed to ease management of limb volume loss are available. There are sockets with panels or struts that the prosthesis user moves radially inward to reduce socket size [2,3], using a mechanical adjustment mechanism on the outside of the socket. Sockets are available that increase limb volume via application of elevated vacuum between the socket and liner, adjusted via a handheld remote-control unit. These technologies eliminate the inconvenience of socket doffing to accommodate limb volume loss but require the user to determine and effect appropriate adjustment—enough to correct the loose socket but not so excessive so as to restrict blood flow or cause further limb volume loss. The cumulative effect of these small decisions can be a significant mental burden, in some cases enough that users prefer not to adjust the socket when offered.

A next step in the development of adjustable sockets is to automate adjustment, thereby reducing distraction and inconvenience and allowing people with limb amputation to live more independent lives. A mechanical system was described in 2003 that adjusted liquid in bladders affixed to the inside socket wall via distal pressure applied to a chamber in the bottom of the socket [4]. Several active systems that adjust socket size based on measurements from interface stress sensors in the socket have been described [5,6,7,8,9]. However, other than one researcher who demonstrated a change in sensed pressure upon activation of the actuator on amputee participants [6], active adjustable sockets using pressure sensing have not advanced into clinical testing.

Inductive sensing is a means for monitoring limb-to-socket distance that is showing promise for use in adjustable socket systems. Distance sensing may be more effective than pressure sensing since it monitors socket looseness, not just how tightly the residual limb is coupled with the socket wall. A “bell clapping” action of the limb in the socket, which may occur during ambulation due to the high sagittal plane bending moment applied during gait [10], is unfavorable since it focuses interface stresses in soft tissues over the tibia, a sensitive area of the residual limb prone to skin injury. As a modality, inductive sensing offers high resolution, stability, simplicity, and immunity to environmental fluctuations.

**Figure 1:**
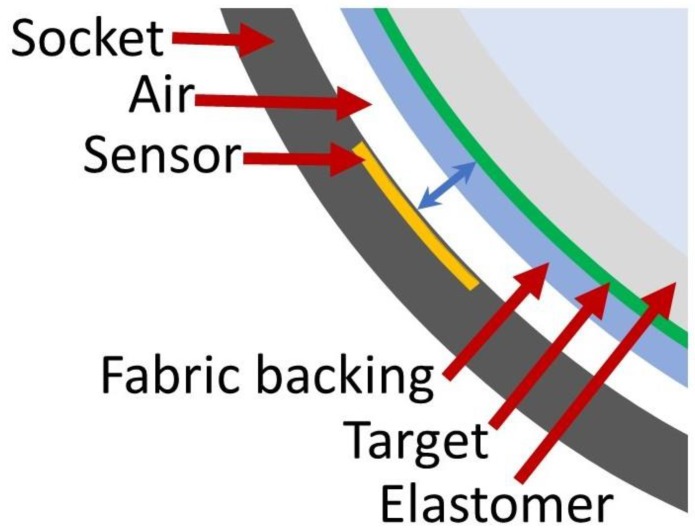
Schematic showing one configuration for inductive sensing in prosthetic sockets. An antenna is positioned in the socket and a target in the liner. In this example there is an air gap between the socket and liner. Distance between sensor and target is indicated with a blue arrow.

Though inductive sensing has been used in a number of health monitoring and home care applications [11], its use in external prosthetics is relatively new. Two groups, at the Ohio Willow Wood Company and the University of Washington, have pursued inductive sensing in prosthetics. Researchers place an antenna within or on the prosthetic socket, and a target material on the user’s liner, [12,13,14,15] (Figure 1). An inductive sensing chip powers the antenna so that the system operates as an LC tank oscillator. Changes in sensor frequency measured by the inductive sensing chip are a sensitive measure of distance between the antenna and target. A change in sensed distance upon prosthetic socket size adjustment and upon application of elevated vacuum has been demonstrated and confirmed using video fluoroscopy [16,17,18,19]. Further, changes in socket fit from sub-optimally sized sockets were detected sooner by an inductive sensor than by an experienced prosthetist visually inspecting participants’ gait [20].

A key to this technology is the inductive sensing target. It must produce sufficient signal intensity and be durable but must also be flexible and lightweight so as to not mechanically traumatize the residual limb. Targets may be conductive or magnetically permeable. Conductive target fabrics affixed to the liner were found to degrade over two weeks from the continual mechanical stresses applied by the socket during user activity [21], thus were unacceptable for extended monitoring. Magnetically permeable targets were proven more durable [22]. An iron powder–polyurethane polymer incorporated into a prosthetic sheath lost less than 2.0% of its signal intensity after four weeks of wear. However, the ferrous polymer sheath was relatively stiff in tension compared to the user’s regular liner, and its 1.0 mm thickness added volume to the socket, which made the socket too tight and thus unusable for some candidate study participants. A thinner ferrous polymer and one of comparable tensile elasticity to elastomeric liners would be less obtrusive and have less detrimental impact on socket fit.

The purpose of this research was to create a magnetically permeable target that integrates into prosthesis users’ liners without affecting liner tensile properties or adding meaningful volume. We sought to create a thin ferrous polymer sheet to be affixed to the outside of the liner elastomer (next to the fabric backing) that was less than 1.00 mm thick, relatively stiff under compressive stresses experienced by amputee prosthesis users so that its performance was minimally sensitive to compression, but at the same time mimicked the tensile properties of existing elastomeric liners (tensile elasticity between 124 and 309 kPa) [23]. The material was to demonstrate minimal signal degradation over at least two weeks of regular use. The design of the material and its performance are presented.

## 2. Materials and Methods

To develop the target material, a polymer substrate was selected, sensor sensitivity to magnetic material concentration and material thickness were tested and optimum values selected, and then material properties, sensitivity, error from applied compression, and durability of the new material were characterized.

We sought to develop a ferrous target made up of a polymer substrate seeded with iron powder. The basis for using iron powder is that it was demonstrated effective previously in development of a ferrous sheath [22]. The sensing element, which may be placed either on or within the wall of the prosthetic socket [19,21], is a coil antenna of diameter 32.0 mm and thickness 0.15 mm made using flexible circuit technology [13]. A surface-mounted capacitor is affixed to the antenna. This sensing element is wired to an inductive sensing chip (LDC1614, Texas Instruments, Dallas, TX, USA) so that when it is powered the inductor and capacitor operate as an LC tank oscillator. The magnetically permeable target near the sensor reinforces the inductor and lowers the sensor’s oscillation frequency in a distance-dependent manner. Therefore, changes in sensor frequency measured by the inductive sensing chip are a sensitive measure of the distance between the antenna and the magnetically permeable target. The sensor is calibrated in the socket using spacers of known thickness [14].

Outside of woven forms, most thermoplastics are not flexible or elastic enough to match the properties of prosthetic liners and thus serve as the polymer substrate. Three potential exceptions are thermoplastic vulcanizates (TPV), thermoplastic polyurethanes (TPU), and thermoplastic elastomers (TPE). TPVs are fully cross-linked ethylene propylene diene terpolymer (EPDM)/polyolefin phase (PP) compounds. TPUs are thermoplastic elastomers consisting of linear segmented block copolymers composed of hard and soft segments. TPEs are copolymers that can be a mixture of polymers. They have both thermoplastic and elastomeric properties. These polymers mimic the mechanical behavior of elastomers while retaining the ability to be heated and molded. TPEs encompass a much broader range of elasticities than the others; TPVs and TPUs fall at the stiff end of the TPE tensile elasticity spectrum. Samples of both TPV and TPU polymers were tested to see if their properties could be modified sufficiently to achieve the range of desired liner elasticity. Preliminary test results showed that at the highest plasticizer loading possible, which would create the lowest stiffness material, samples from both TPV and TPU groups remained beyond the high end of the stiffness range of most elastomeric liners (tensile stiffness >309 kPa) [23]. Thus, TPV and TPU materials were not considered further, and TPEs were selected for development in the present study.

Three TPEs were considered. The first material, 4044, is a SEEPS (styrene–ethylene–ethylene/propylene–styrene) polymer. The second material, 2063, is a SPS (styrene–propylene–styrene) polymer. The third material, an alloy, is made from two different SBS (styrene–butylene–styrene) polymers. The styrene content of each polymer and the average molecular weight of the chains are the most influential factors that determine polymer strength. Different midblock chemistries affect the amount of plasticizer the polymer can absorb and thus its elasticity, but of equal importance a variety of chemistries provide a good chance of finding a suitable adhesive for bonding the target to the elastomer or fabric backing of a typical prosthetic liner product. Preliminary testing demonstrated 4044 the most promising of these three polymers in that it demonstrated compressive and tensile elasticities within the desired range for this application.

**Figure 2:**
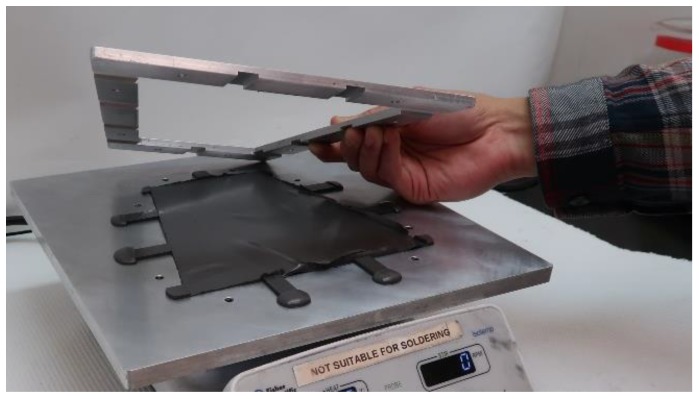
Ferrous polymer construct being removed from its mold.

Ferrous target samples of thickness ranging from 0.50 to 1.00 mm were fabricated for testing. To make the samples, light mineral oil was mixed with Septon 4044 (Kurarary America, Pasadena, TX, USA), placed on a hotplate, and stirred until it congealed. Iron powder was added, the material mixed, and then placed into the base of a mold (coated with a release agent) that had venting channels (Figure 2). Three molds were used, of heights 0.50, 0.75, and 1.00 mm. A top plate was loaded with weights to compress the viscous polymer so that excess polymer was forced out through the venting channels. The mold was allowed to cool, and the material was removed and cut into 50.8 mm diameter samples for testing.

A high iron concentration compared with a low iron concentration will induce a stronger signal from the inductive sensor but will increase cost and may also increase the tensile elasticity of the target. In prior efforts developing a polyurethane-based target within a prosthetic sheath fabric, we found that a concentration of 75% to 83% produced an appropriate sensitivity [22]. In the present study using the TPE polymer, three iron concentrations were evaluated (75%, 77%, and 85% by weight). It was expected that the highest concentration (85%) would approach saturation of the signal, thus experience minimal error from compressive stress compared with the lower concentrations.

**Figure 3:**
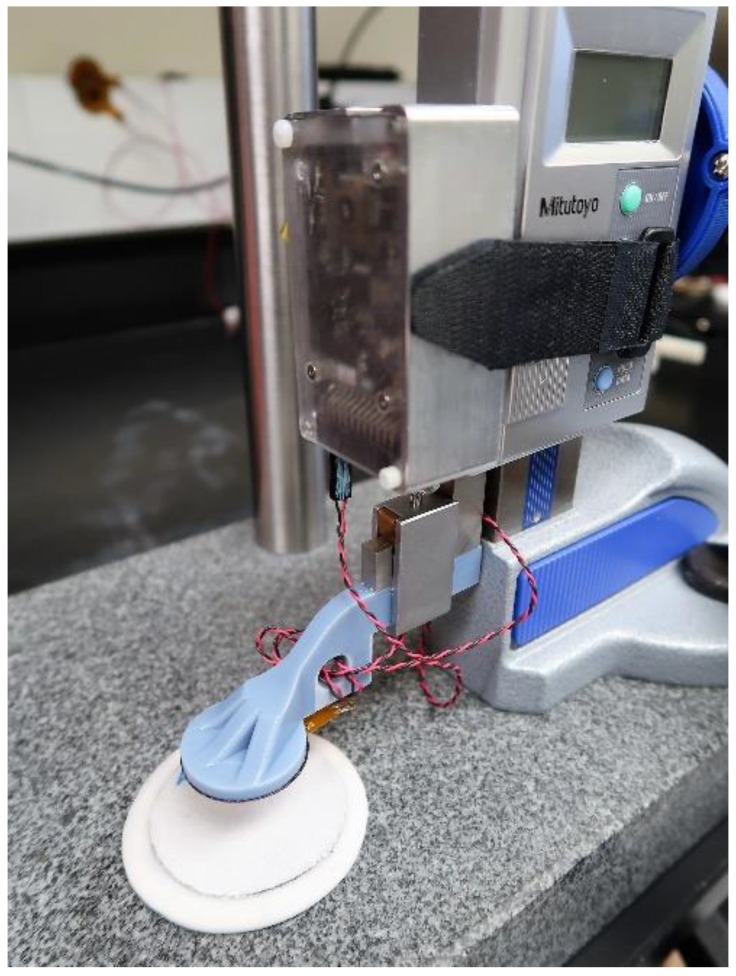
Testing jig for the evaluation of sensitivity. The sensor antenna is mounted within the arm connected to the height gauge. The sample material is affixed to the table.

To characterize sensitivity of the inductive sensor to targets of different iron concentration, a testing jig was used (Figure 3). The jig allowed the inductive sensor antenna to be positioned above a target sample at a known height, recorded using a height gauge (570–312, Mitutoyo, Aurora, IL, USA) with resolution of 0.01 mm. The antenna was adjusted in incremental steps, 0.25 mm for distances up to 2.00 mm, and 1.00 mm for distances between 2.00 mm and 15.00 mm.

A thinner magnetic target is preferable since it occupies less volume in the socket and is thus less disruptive to prosthesis users. Thicknesses of 0.50, 0.75, and 1.00 mm were tested.

To characterize sensitivity of the inductive sensor to target thickness, the testing jig shown in Figure 3 was used. Data points were concentrated at low distance so as to obtain an accurate sensitivity reading in the high sensitivity region of the calibration curve and to ensure the 0.00 mm distance was well defined. The antenna was adjusted in incremental steps, 0.05 mm for distances up to 0.60 mm, 0.25 mm for distances between 0.60 mm and 2.00 mm, and 1.00 mm for distances above 2.00 mm but less than 15.00 mm.

Compression testing was conducted using procedures similar to those described previously for testing prosthetic liners [23]. A material testing machine was used (Instron 5944, Norwood, MA, USA). Samples were of dimension 19.0 mm diameter. Synthetic lubricant (Outlast, C & C Synthetics, Mandeville, LA, USA) was applied to the target material samples to reduce friction. Samples were cyclically preconditioned for 15 minutes from 0 to 250 kPa at a rate of 100% strain per second and then re-lubricated before the test. They were then compressed to 60% strain at a rate of 150% strain per second. Tests were conducted until two cycles of repeatable stress strain response were achieved.

Measured engineering stress was converted to true stress under the assumption of material incompressibility. Compressive elasticity (CE) was calculated as the tangent modulus between 10% and 40% strain [23]. Three samples were tested, and the overall CE was the arithmetic mean of the three of them.

Tensile testing was conducted using procedures similar to those described previously for testing prosthetic liners [23]. Samples of target material of dimension 170.0 mm × 31.3 mm were adhered at the ends of a custom test fixture equipped with linear slide rails to accommodate the changing thickness in the sample during the test. The two halves of the jig were installed using parallel blocks to ensure the sample was properly aligned. The sample was pulled at a displacement rate of 30 mm/s until a 60% strain was achieved. The sample was allowed to rest for 20 minutes and then the test was rerun. Testing was repeated until two consistent results were achieved.

Engineering stress was converted to true stress using the assumption of incompressibility. From the resulting stress strain plot, the slope of the tangent line to the curve between strains of 10% and 40% was calculated. The slope of this line was the tensile elasticity for each sample. Three samples were tested, and the overall tensile elasticity was the arithmetic mean of the three of them.

Volumetric elasticity testing was conducted similar to that described previously for liner testing [23]. Samples 10.0 mm in diameter and 1.0 mm thick were coated with synthetic lubricant and placed in a 10.0 mm diameter well. Six samples were stacked in the well to achieve a thickness comparable to prosthetic liners tested previously (approximately 6.0 mm). The plunger of the tensile testing machine was lowered into the well and compressed the specimen at 0.1 percent strain per second up to 500 kPa compression. Tests were conducted until two cycles of repeatable stress strain response were achieved. Using the highest loads tested, the line tangent to the linear region was calculated and the slope reported as the volumetric elasticity (VE).

Using the VE and CE values for each sample, we calculated the Poisson ratio using Hooke’s law and the definition of Poisson ratio:

(1) $$PR=\frac{3*VE-CE}{6*VE}$$

**Figure 4:**
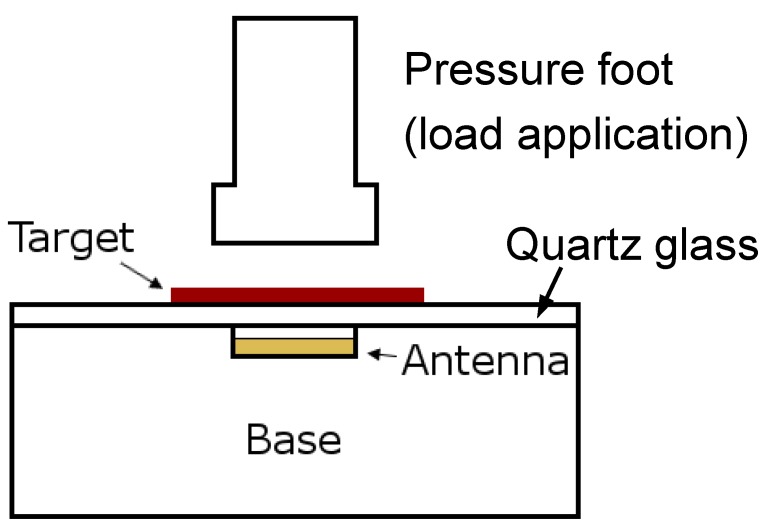
Schematic of testing configuration to evaluate response under compressive stress.

Evaluations were conducted to characterize sensor sensitivity to compressive stress. A base with a cutout space was used to isolate the antenna and keep it at a consistent distance from the target (2.0 mm) during testing (Figure 4). A 1.6 mm-thick plate of quartz glass (elasticity of 72 GPa) was placed on top of the base to minimize deformation of the support jig. Testing machine load and displacement data (Instron 5944, Norwood, MA, USA) were collected simultaneously with sensor data.

A 50.8 mm diameter target material sample was centered above the antenna. Preliminary testing demonstrated that samples tended to stick to the platen during testing, thus a thin layer of synthetic lubricant was applied to the sample. Six pre-conditioning cycles from 10 kPa to 425 kPa were applied. The crosshead rate was 30 mm/s during loading and unloading. A pressure of 425 kPa was held for 15 s during each cycle. Addition of the lubricant introduced a “toe” region to the material property curve at pressures below 25 kPa. Lubricant was squeezed out from between the platen and test sample. Therefore, after preconditioning, the pressure was stepped from 25 kPa to 425 kPa in 40 kPa increments and held for 15 s at each pressure.

**Figure 5:**
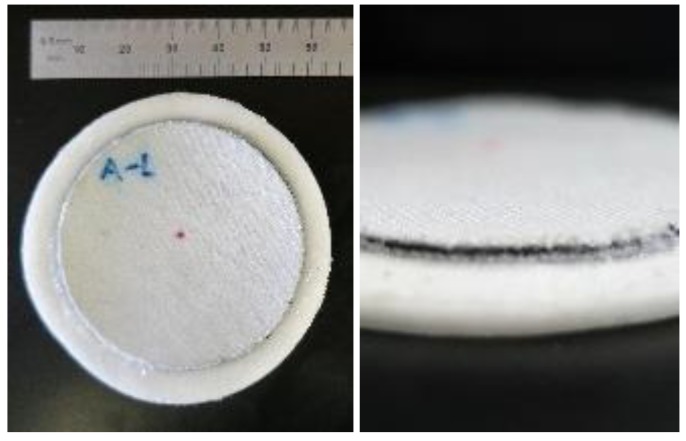
Sample ready for durability testing.

Durability testing was conducted using a fabric–polymer–fabric stack-up so that the 0.50 mm-thick polymer target did not fold over itself when the test sample was worn in the shoe of an able-bodied person. The stack-up was made in a two-step process. First, a 0.50 mm-thick sample of the ferrous polymer was overlaid onto a 0.60 mm-thick sheet of nylon spandex (FLNS, Seattle Fabrics, Inc., Seattle, WA, USA). This construct was placed in a mold and heated to mechanically bond together. This process was repeated on the opposite surface with a second layer of fabric so as to sandwich the 0.50 mm-thick ferrous polymer sample between the two fabric sheets. Samples of 50.8 mm diameter were punched out of the finished stack-up. These samples were affixed to the silicone elastomer from a prosthetic liner using silicone adhesive (Sil-Poxy, Smooth-On, Inc., Macungie, PA, USA). The elastomer from the liner was 63.5 mm in diameter and 2.30 mm in thickness (Figure 5).

One testing sample was worn in each shoe under the metatarsal region of the foot by a 65.7 kg male for 14 days. A smart phone step counter was used to monitor the number of steps during the test period. Over 14 days, the mean step count was 2,123 steps/day. Calibration tests were performed before the sample was worn (pre-wear) and were repeated at 1d, 3d, 7d, 10d and 14d of wear. The elastomeric liner material affixed to the sample plastically compressed over time, which was accounted for in calibration by shifting the zero position of the sample by the reduced thickness.

## 3. Results

**Figure 6:**
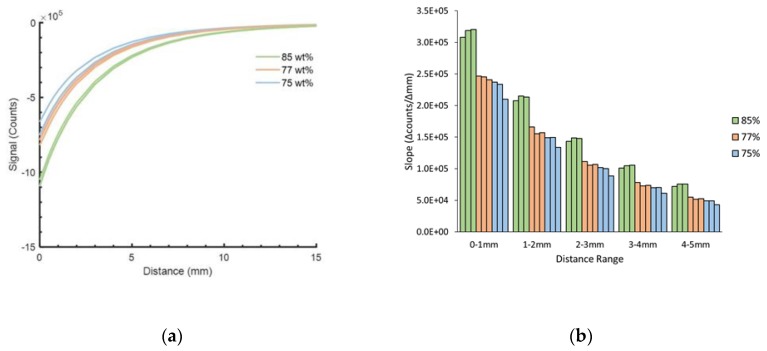
Iron concentration test results. (**a**) Calibration test results for three samples at each iron concentration are shown. (**b**) Slope of calibration curve (Δcounts/Δmm) at different distance ranges from the target for constructs with different iron concentrations. Three samples were tested for each concentration—85 wt%, 77 wt%, and 75 wt%. 1.00 mm thickness samples. (Supplementary Materials)

For 1.00 mm-thick samples, material of 85 wt% demonstrated higher sensitivity (counts/mm) than material of 77 wt% or 75 wt% (Figure 6a). On average, at distances less than 5.0 mm, the 85 wt% sample had 1.33 times the sensitivity of the 77 wt% sample and 1.46 times the sensitivity of the 75 wt% sample. Sensitivities decreased as distance from the target increased (Figure 6b).

**Figure 7:**
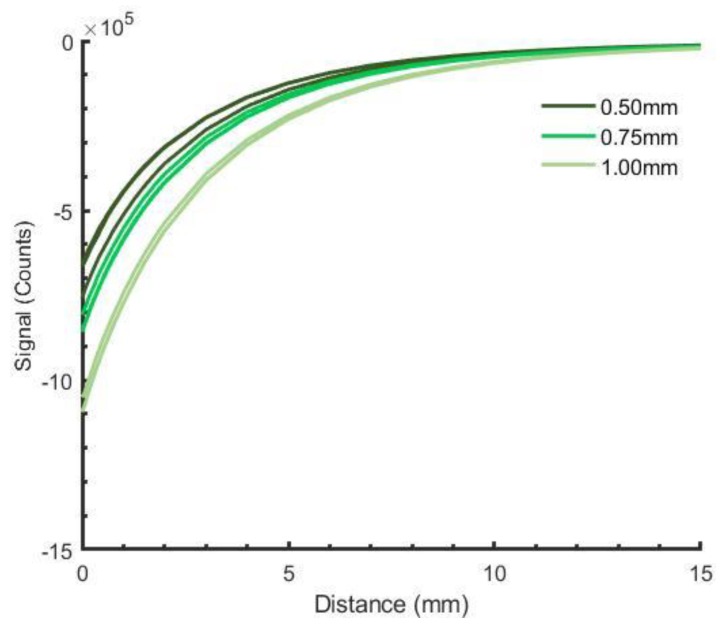
Performance of 85 wt% constructs of varying thickness. Results for each of three samples at each thickness are shown. (Supplementary Materials)

Sensitivity (counts/mm) increased with thickness (Figure 7). Sensitivity of the 0.50 mm construct at distances less than 5.00 mm averaged 0.84 times that of 0.75 mm constructs and 0.66 times that of 1.00 mm constructs.

CE increased with increased iron concentration. Median compressive elasticity of 1.00-mm thick samples was 421 kPa for 85 wt% samples, 238 kPa for 77 wt% samples, and 224 kPa for 75 wt% samples.

Because of its acceptable sensitivity, low volume, and high CE compared with the other designs, the 0.50 mm-thick, 85 wt% iron concentration design was selected. Material testing of 0.50 mm samples demonstrated a compressive elasticity of 418 to 487 kPa. Tensile elasticity ranged from 267 to 313 kPa (Table 1).

For samples of 0.50 mm thickness with iron concentration of 85 wt%, sensed distance decreased 2.9 μm for applied pressures between 25 kPa and 100 kPa. For pressures between 100 kPa and 425 kPa, sensed distance changed by 0.41 μm, which is less than the bit resolution of the data acquisition system.

**Figure 8:**
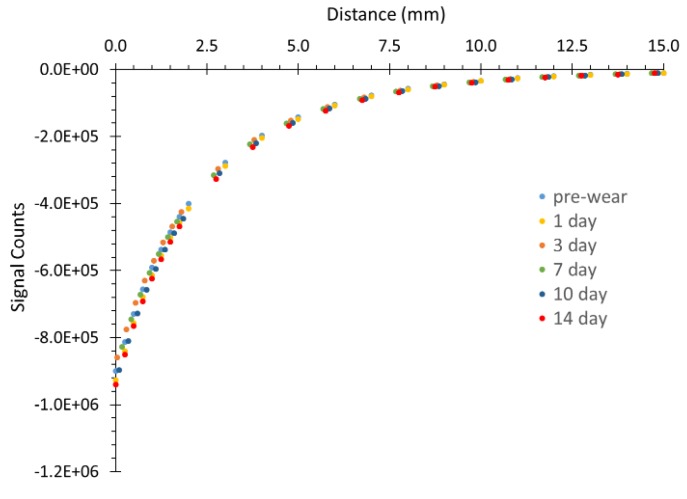
Durability test results for different wear times. (Supplementary Materials)

Durability testing demonstrated consistent target performance over two weeks of wear (Figure 8). The analysis of the data showed that the coefficient of variation (standard deviation/mean) between pre-wear and 14 d wear durations was lower for the mid-distance ranges (2.8% for 1.00–2.00 mm and 3.00–4.00 mm; 3.1% for 2.00–3.00 mm) than the low or high distance ranges (3.8% for 0.00–1.00 mm; 4.1% for 4.00–5.00 mm). There was not a trend of decreased or increased sensitivity with longer wear duration.

## 4. Discussion

Measurement of distance between the residual limb and socket may be useful in automatically adjusting prosthetic sockets for people with limb loss. Ideally, socket size would adjust to maintain fit (sensed distance) despite changes in limb volume during ambulation. The sensing system described here monitors only perpendicular distance between the limb and socket and no other types of motion (e.g., tangential). This is a beneficial feature in this application because perpendicular motion relates directly to limb volume while motions in other directions do not. Changes in socket fit due to changes in limb volume are the primary source of socket fit issues for people using prosthetic limbs [1].

A novel ferrous polymer to serve as a magnetically permeable target for inductive distance sensing was developed in this research. The construct (0.50 mm thickness, 85 wt%) decreased socket volume available to the residual limb by approximately 2.1%, (using Fernie’s residual limb model [24]). Tensile elasticity (267 to 313 kPa) was comparable to that of existing liners (124 to 309 kPa) thus the construct introduces minimal material property mismatching when adhered to an existing liner product. In concept, the tensile stiffness of the ferrous polymer could be tuned to match that of any liner manufacturer’s elastomer by adjusting the plasticizer concentration.

The SEEPS TPE developed here is more elastic under tension than the polyurethane fabric sheath developed previously [22] because it can absorb a higher concentration of plasticizer, allowing the polymer chains to easily rearrange themselves under tension. The plasticizer takes up volume so that the polymer chains are not as densely packed. TPEs have long mid-blocks that are normally compacted but are stretched when they are pulled, also contributing to improved tensile elasticity.

As expected, sensor sensitivity increased with increased iron concentration and decreased with decreased thickness, indicating that signal intensity was sensitive to the amount of iron present. Higher iron concentrations may be possible which would be expected to saturate the polymer and produce an even more constant signal for varying thickness samples. However, the error under applied compressive stress was low (2.9 μm for pressures between 25 and 100 kPa, and ≤0.41 μm for pressures between 100 and 425 kPa). This characteristic was in part due to the construct’s relatively high compressive stiffness (418 to 487 kPa). Further, signal sensitivity was expected adequate to meet the needs of prosthetics application, and considering the expense of iron powder, additional efforts to enhance iron concentration were deemed unnecessary.

A next step in this effort will be to adhere the ferrous polymer target to prosthetic liners of participants with limb amputation and evaluate its capability to monitor socket fit over time in free-living environments of people with limb amputation.

## Figures and Tables

**Table 1:** Material properties of developed construct compared with commercial elastomeric liners.

Material Property	Construct (85 wt%, 0.50 mm-Thick) (Median (Range))	Elastomeric Liners (Range from [23])
Compressive Elasticity (kPa)	448 (418–487)	96–458
Tensile Elasticity (kPa)	282 (267–313)	124–309 *
Poisson Ratio	0.4947 (0.4945–0.4949)	0.4929–0.4999

* Only for liners whose tensile stiffness was not dominated by its fabric backing material, as described in [23].

## Supplementary Materials

Data are available online at https://www.mdpi.com/1424-8220/19/18/4041/s1.

## References

[1] Legro M.W., Reiber G., Del Aguila M., Ajax M.J., Boone D.A., Larsen J.A., Smith D.G., Sangeorzan B. (1999). Issues of importance reported by persons with lower limb amputations and prostheses. J. Rehabil. Res. Dev..

[2] Wilson A.B., Schuch C.M., Nitschke R.O. (1987). A variable volume socket for below-knee prostheses. Clin. Prosthet. Orthot..

[3] Kahle J.T., Klenow T.D., Highsmith M.J. (2016). Comparative effectiveness of an adjustable transfermoral prosthetic interface accommodating volume fluctuation: case study. Technol. Innov..

[4] Greenwald R.M., Dean R.C., Board W.J. (2003). Volume management: Smart Variable Geometry Socket (SVGS) technology for lower limb prostheses. J. Prosthet. Orthot..

[5] Mercier M., Shirley C., Stafford S., Hitzke S., Byju A., Kevorkian C., Madigan M., Philen M. Fluidic flexible matrix composites for volume management in prosthetic sockets. Proceedings of the ASME 2014 Conference on Smart Materials, Adaptive Structures and Intelligent Systems.

[6] Pirouzi G., Abu Osman N.A., Oshkour A.A., Ali S., Gholizadeh H., Abas W.A.B.W. (2014). Development of an Air Pneumatic Suspension System for Transtibial Prostheses. Sensors.

[7] Wheeler J.W., Mazumdar A., Marron L.C., Dullea K.J., Sanders J., Allyn K. Development and amputee validation of pressure and shear sensing liner for prosthetic sockets. Proceedings of the IEEE Engineering in Medicine and Biology Society.

[8] Candrea D., Sharma A., Osborn L., Gu Y., Thakor N. An adaptable prosthetic socket: Regulating independent air bladders through closed-loop control. Proceedings of the 2017 IEEE International Symposium on Circuits and Systems (ISCAS).

[9] Carrigan W., Nothnagle C., Savant P., Gao F., Wijesundara M.B.J. Pneumatic actuator inserts for interface pressure mapping and fit improvement in lower extremity prosthetics. Proceedings of the IEEE RAS/EMBS International Conference on Biomedical Robotics and Biomechatronics.

[10] Radcliffe C.W. (1962). The biomechanics of below-knee prostheses in normal, level, bipedal walking. Artif. Limbs.

[11] Tang X., Miao Y., Chen X., Nie B. (2019). A Flexible and Highly Sensitive Inductive Pressure Sensor Array Based on Ferrite Films. Sensors.

[12] Wernke M., McGough J., Albury A., Denune J., Doddroe C., Colvin J., Rink C., Sen C., Hendershot B., Dearth C. Multiaxial in-socket movement and its relationship to fit. Proceedings of the American Academy of Orthotists & Prosthetists 44th Academy Annual Meeting and Scientific Symposium.

[13] Swanson E.C., McLean J.B., Allyn K.J., Redd C.B., Sanders J.E. (2018). Instrumented socket inserts for sensing interaction at the limb-socket interface. Med. Engi. Phys..

[14] Henrikson K.M., Weathersby E.J., Larsen B.G., Cagle J.C., McLean J.B., Sanders J.E. (2018). An Inductive Sensing System to Measure In-Socket Residual Limb Displacements for People Using Lower-Limb Prostheses. Sensors.

[15] McLean J.B., Redd C.B., Larsen B.G., Garbini J.L., Brzostowski J.T., Hafner B.J., Sanders J.E. (2019). Socket size adjustments in people with transtibial amputation: Effects on residual limb fluid volume and limb-socket distance. Clin. Biomech..

[16] Gerschutz M.J., Hayne M.L., Colvin J.M., Denune J.A. (2015). Dynamic Effectiveness Evaluation of Elevated Vacuum Suspension. JPO J. Prosthetics Orthot..

[17] Weathersby E.J., Garbini J.L., McLean J.B., Vamos A.C., Sanders J.E. (2019). Automatic control of adjustable prosthetic sockets: Implementation and evaluation. IEEE Trans. Biomed. Engi..

[18] Darter B.J., Sinitski K., Wilken J.M. (2016). Axial bone-socket displacement for persons with a traumatic transtibial amputation: The effect of elevated vacuum suspension at progressive body-weight loads. Prosthetics Orthot. Int..

[19] Larsen B.G., McLean J.B., Allyn K.J., Brzostowski J.T., Garbini J.L., Sanders J.E. (2019). How do transtibial residual limbs adjust to intermittent incremental socket volume changes?. Prosthet. Orthot. Int..

[20] Larsen B.G., Allyn K.J., Ciol M.A., Sanders J.E. (2019). Performance of a sensor to monitor socket fit: Comparison with practitioner clinical assessment. J. Prosthet. Orthot..

[21] Sanders J.E., Redd C.B., Larsen B.G., Vamos A.C., Brzostowski J.T., Hafner B.J., Allyn K.J., Henrikson K.M., McLean J.B., Hinrichs P. (2018). A Novel Method for Assessing Prosthesis Use and Accommodation Practices of People with Transtibial Amputation. JPO J. Prosthet. Orthot..

[22] Weathersby E.J., Cagle J.C., Larsen B.G., Henrikson K.M., Sanders J.E. (2018). Development of a magnetic composite material for measurement of residual limb displacements in prosthetic sockets. J. Rehabil. Assist. Technol. Eng..

[23] Cagle J.C., Hafner B.J., Taflin N., Sanders J.E. (2018). Characterization of Prosthetic Liner Products for People with Transtibial Amputation. JPO J. Prosthet. Orthot..

[24] Fernie G.R., Holliday P.J. (1982). Volume fluctuations in the residual limbs of lower limb amputees. Arch. Phys. Med. Rehabil..

